# Increased cognitive workload evokes greater neurovascular coupling responses in healthy young adults

**DOI:** 10.1371/journal.pone.0250043

**Published:** 2021-05-19

**Authors:** Tamas Csipo, Agnes Lipecz, Peter Mukli, Dhay Bahadli, Osamah Abdulhussein, Cameron D. Owens, Stefano Tarantini, Rachel A. Hand, Valeriya Yabluchanska, J. Mikhail Kellawan, Farzaneh Sorond, Judith A. James, Anna Csiszar, Zoltan I. Ungvari, Andriy Yabluchanskiy

**Affiliations:** 1 Vascular Cognitive Impairment and Neurodegeneration Program, Center for Geroscience and Healthy Brain Aging, Department of Biochemistry and Molecular Biology, University of Oklahoma Health Sciences Center, Oklahoma City, Oklahoma, United States of America; 2 Department of Public Health, Semmelweis University, Faculty of Medicine, Budapest, Hungary; 3 Department of Ophthalmology, Josa Andras Hospital, Nyiregyhaza, Hungary; 4 Department of Physiology, Semmelweis University, Faculty of Medicine, Budapest, Hungary; 5 Bon Secours St. Francis Family Medicine Center, Midlothian, Virginia, United States of America; 6 Department of Health and Exercise Science, University of Oklahoma, Norman, Oklahoma, United States of America; 7 Division of Stroke and Neurocritical, Department of Neurology, Northwestern University Feinberg School of Medicine, Chicago, Illinois, United States of America; 8 Arthritis & Clinical Immunology Research Program, Oklahoma Medical Research Foundation, Oklahoma City, Oklahoma, United States of America; 9 Department of Medical Physics and Informatics, University of Szeged, Szeged, Hungary; 10 Department of Health Promotion Sciences, College of Public Health, University of Oklahoma Health Sciences Center, Oklahoma City, Oklahoma, United States of America; 11 Section of Geriatrics, Department of Internal Medicine, University of Oklahoma Health Sciences Center, Oklahoma City, Oklahoma, United States of America; Tokyo Joshi Ika Daigaku Toyo Igaku Kenkyujo Clinic, JAPAN

## Abstract

Understanding how the brain allocates resources to match the demands of active neurons under physiological conditions is critically important. Increased metabolic demands of active brain regions are matched with hemodynamic responses known as neurovascular coupling (NVC). Several methods that allow noninvasive assessment of brain activity in humans detect NVC and early detection of NVC impairment may serve as an early marker of cognitive impairment. Therefore, non-invasive NVC assessments may serve as a valuable tool to detect early signs of cognitive impairment and dementia. Working memory tasks are routinely employed in the evaluation of cognitive task-evoked NVC responses. However, recent attempts that utilized functional near-infrared spectroscopy (fNIRS) or transcranial Doppler sonography (TCD) while using a similar working memory paradigm did not provide convincing evidence for the correlation of the hemodynamic variables measured by these two methods. In the current study, we aimed to compare fNIRS and TCD in their performance of differentiating NVC responses evoked by different levels of working memory workload during the same working memory task used as cognitive stimulation. Fourteen healthy young individuals were recruited for this study and performed an *n*-back cognitive test during TCD and fNIRS monitoring. During TCD monitoring, the middle cerebral artery (MCA) flow was bilaterally increased during the task associated with greater cognitive effort. fNIRS also detected significantly increased activation during a more challenging task in the left dorsolateral prefrontal cortex (DLPFC), and in addition, widespread activation of the medial prefrontal cortex (mPFC) was also revealed. Robust changes in prefrontal cortex hemodynamics may explain the profound change in MCA blood flow during the same cognitive task. Overall, our data support our hypothesis that both TCD and fNIRS methods can discriminate NVC evoked by higher demand tasks compared to baseline or lower demand tasks.

## Introduction

Under cognitive workload, activation of neurons in the brain implies the allocation of resources and nutrients by the cerebrovascular system. A better understanding of this physiological adaptation may aid timely identification of individuals in early stages of cognitive impairment, provided that a neuroimaging modality is available with adequate sensitivity to detect functional responses elicited by cognitive stimuli. Early interventions would allow improvements in healthspan and postpone deterioration of quality of life in individuals prone to dementia.

Under physiological conditions, the brain is critically dependent on the structural and functional integrity of its blood vessels. To supply additional nutrients to match the metabolic demand of the cells in the central nervous system, activation of the neuronal population in the brain is accompanied by functional hyperemia, also known as neurovascular coupling (NVC). In human subjects, impaired NVC is associated with neurological diseases (stroke, Alzheimer’s disease) that lead to cognitive impairment [1]. Interestingly, recent studies suggested that decreased cognitive performance may be associated with increased or decreased cortical NVC responses during certain cognitive tasks [2, 3], depending on the type and difficulty of the task. These recent developments highlight the importance of further investigations of NVC to better understand the resource allocation of the brain while performing cognitive tasks under physiological conditions.

A relatively ubiquitous method, Transcranial Doppler sonography (TCD) allows rapid and economical assessment of dynamic blood flow regulation in the brain. Specific task-evoked NVC can be assessed during a cognitive task (e. g., a working memory paradigm with different levels of difficulty) while monitoring blood flow in the middle cerebral artery (MCA) [4]. However, TCD methodology does not allow monitoring of blood vessel diameters. It is assumed that MCA diameter remains constant during testing and that increases in the blood flow velocities are proportional to increases in blood flow caused by the dilation of arterioles downstream TCD offers low spatial resolution as it can only measure NVC in major supply arteries of the brain, therefore, it cannot localize activation to a specific area of the brain.

NVC can also be assessed by functional Magnetic Resonance Imaging (fMRI) [5]. During fMRI, the production and washout of paramagnetic deoxy-hemoglobin (HbR) represent a focal change of blood flow around activated brain regions which creates a signal that is commonly referred to as the blood oxygen level-dependent (BOLD) signal [6]. When interpreting fMRI results, NVC is commonly considered a marker of neuronal activation and the vascular component of NVC is often not considered, however, the captured BOLD signal originates from the microcirculation of the brain.

Functional near infrared spectroscopy (fNIRS) is a relatively new method that allows a more economical assessment of NVC [7] in the microcirculation of the cerebral cortex. In contrast to fMRI, which only allows for measurement of HbR, it is capable of measuring changes both in oxy-hemoglobin (HbO) and HbR concentrations. Although fNIRS underperforms fMRI in its spatial resolution in the cortex, the technique provides satisfactory resolution for applications in functional neuroimaging studies. fNIRS illuminates tissue with multiple wavelengths of light from the near-infrared spectrum, which can penetrate through the scalp and skull to reach superficial layers of the cerebral cortex [7]. The light is then partially absorbed by HbO and HbR present in the illuminated tissue, allowing for measurement of the absolute or relative concentration of these chromophores [8]. fNIRS has proven to be a useful tool to detect deactivation in the prefrontal cortex (PFC) in patients with posttraumatic stress disorder while performing a cognitive task [9] or detecting the recruitment of the dorsolateral prefrontal cortex (DLPFC) when a more challenging task was anticipated [10]. Further, results from fNIRS studies correlate well with those done with fMRI methodology [11]. For example, cognitive workload-dependent activation in DLPFC was observed when using fMRI during *n*-back cognitive stimulation [12] and gradually increased activation in the left DLPFC was reported during a similar stimulation paradigm measured with fNIRS [11, 13]. Attempts using a standardized *n*-back cognitive stimulation of several difficulty levels found increased activation in the left DLPFC region with increased task difficulty [13].

The studies mentioned above utilized a working memory paradigm as cognitive stimulation to evoke NVC response [4, 12, 13]. Working memory can be examined via the *n*-back approach, originally published by W. K. Kirchner [14], and this cognitive domain is responsible for temporarily holding information available for manipulating, processing, including critical thinking, solving problems, interacting with others.

Previous studies have demonstrated that NIRS is sensitive to cognitive workload while performing an *n*-back working memory paradigm [15, 16]. However, the reported localized activation of the PFC—typically in the area of the DLPFC—may not explain the bilateral vascular response that can be seen during TCD monitoring while utilizing the same paradigm for cognitive stimulation [4]. In the current study, we expanded the area of measurement during NIRS examinations to capture hemodynamic signals over other cerebral regions. In this study, we aimed to investigate whether (1) activation of other areas in the prefrontal cortex can be measured with NIRS, (2) whether these areas are only activated at higher cognitive workload, (3) and compare the sensitivity of TCD and fNIRS in their capabilities of detecting cognitive states evoked by different levels of cognitive workload during a working memory task. To achieve these aims, an *n*-back working memory paradigm was utilized as cognitive stimulation to evoke NVC response. The cognitive stimulation paradigm consisted of several difficulty levels, and fNIRS was utilized to measure NVC in the PFC (not limited to DLPFC). TCD was used to measure hemodynamic changes in the upstream MCA during the same cognitive task in young, healthy individuals.

## Materials and methods

### Study design and subject characteristics

Study participants were recruited between February 2018 and July 2019 from the employees of the University of Oklahoma Health Sciences Center. Study participants received information about the study via e-mail, over the phone or in person. The whole study was performed in the Translational Geroscience Laboratory of the Center for Geroscience and Healthy Brain Aging, Department of Biochemistry and Molecular Biology, University of Oklahoma Health Sciences Center. A total of fourteen healthy adults (2 left-handed participants, 10 males and 4 females) were enrolled in this study (age: 31±5.94 years, BMI: 24.9±2.95, systolic blood pressure: 116±10.48, diastolic blood pressure: 75±10.47, reported values are mean±SD). None of the participants reported neurological, psychiatric diseases, or any other significant medical condition (cardiovascular disease, cancer, diabetes, infection within 2 weeks of the examination date). Two female subjects reported the use of oral contraceptive medication during the study, and other subjects reported no chronic use of prescribed or over-the-counter medications. No smokers were included in the study. All participants were asked to refrain from consuming caffeinated beverages at least 6 hours prior to the assessments. Four participants had college degrees and 10 participants had doctoral degrees. The study required two visits, and participants were randomized to start with either the TCD or fNIRS assessments during the first visit. The average time between the two assessments was 121.86±77.99 days, and subjects performed the remaining other assessment during the second visit (either fNIRS or TCD) using the same cognitive stimulation paradigm.

Written informed consent was obtained by study personnel from all participants prior to participation in the study. The protocol was approved by the Institutional Review Board of the University of Oklahoma Health Sciences Center and all methods were carried out in accordance with relevant guidelines and regulations.

### Cognitive stimulation

**Fig 1:**
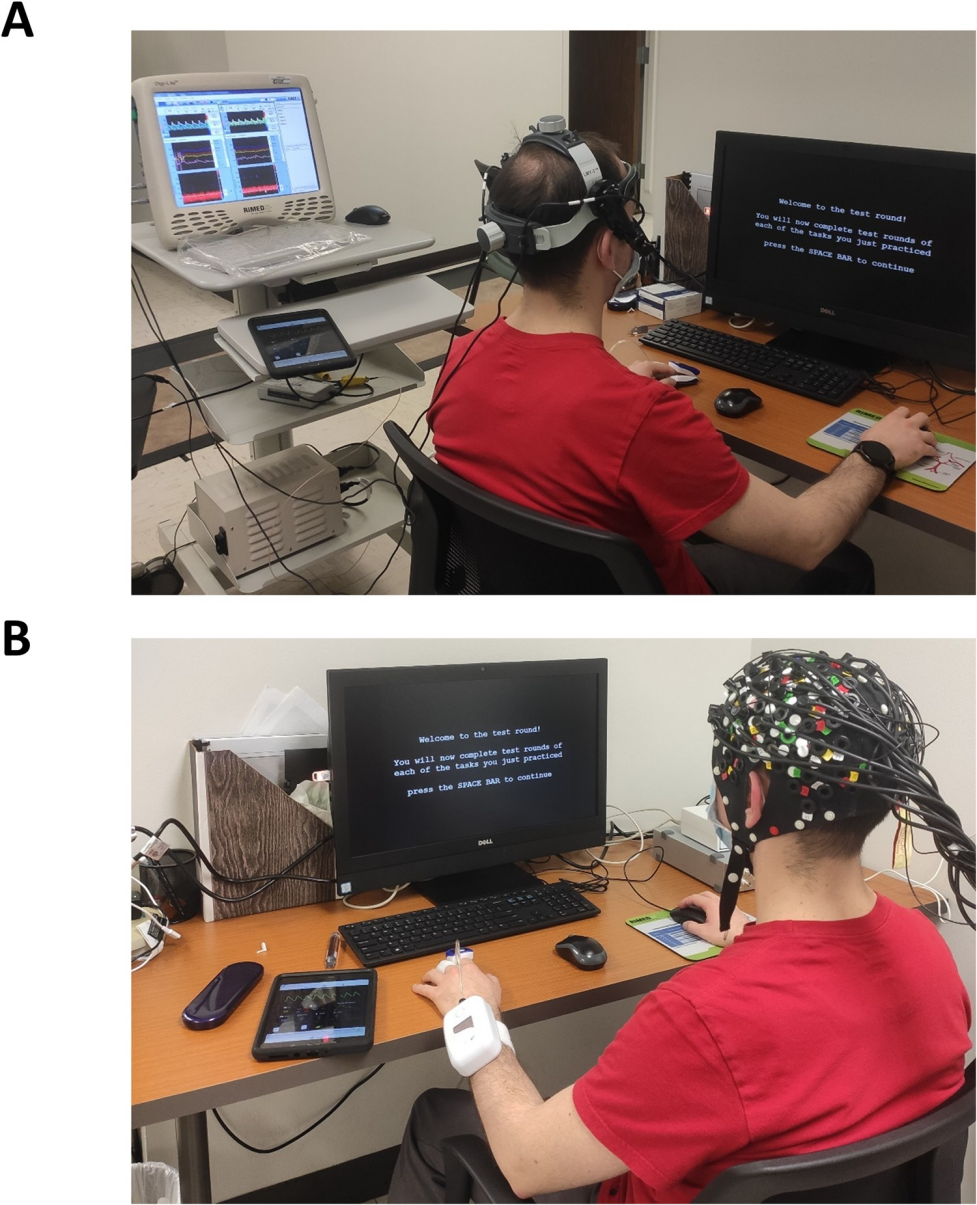
Experimental setup. Participants were seated in front of the computer that delivered the cognitive paradigm. Changes in cerebral hemodynamics were recording during cognitive stimulation with transcranial Doppler sonography (Panel A) and functional near-infrared spectroscopy (Panel B).

In this study, we used an *n*-back paradigm-based cognitive test to evoke NVC responses, as previously described [4]. In brief, participants were seated comfortably in front of a 22-inch monitor, with their right hand resting on the computer mouse (Fig 1). Tasks were first explained in detail before the trials were started. Participants were encouraged to perform as well as they could. During cognitive testing, all participants were presented with the instructions on the monitor screen, and then a white fixation rectangle appeared on a black background. Sixty letters were presented within the fixation rectangle in a random order, each for 250 ms. Participants were presented with three tasks:

1. 0-back or identify W: a click of a mouse button was requested whenever the letter ‘W’ was shown within the fixation rectangle.
2. 1-back: the task was to identify repeated letters in the sequence (e. g. x-y-A-A)
3. 2-back: the task was to identify patterns where every other letter was repeated (e. g. x-A-y-A)

Tasks were administered in the following order: 0-back→ 1-back→ 0-back→ 2-back, during both visits. The interval between presented letters varied from 1850 ms to 2050 ms and was randomized by the custom software developed in ePrime 3 (Psychology Software Tools, Sharpsburg, PA). The average block length was 134.13 ± 1.37 s.

Further, cognitive performance during *n*-back tasks were evaluated by calculating the percentage of correct responses (%correct) and reaction time for each subject, *n*-back task, and each visit.

### Neurovascular coupling protocol with transcranial Doppler (TCD)

MCA flow velocity (MCAv) was assessed using transcranial Doppler sonography (Digi-Lite, Rimed, Raanana, Israel) by placing 2 Mhz ultrasound probes over the left and right temporal acoustic windows. The ultrasound probes were mounted on a probe fixation device (LMY-2, Rimed, Raanana, Israel) that allowed MCAv monitoring at a constant angle for an extended period of time. Left and right MCAv signals were identified according to the standardized criteria guided by signal depth and velocity [17, 18]. After establishing optimal MCAv signal, the probes were secured, and the signal depth and power remained constant throughout the test session. Channels with an inadequate temporal acoustic window or flow velocity were excluded from the analysis. Blood pressure was measured via sphygmomanometry at the beginning of the session, and values were used to calibrate a non-invasive continuous blood pressure monitor (Caretaker4, Caretaker Medical, Charlottesville, VA, USA).

MCA flow velocity envelope was continuously recorded with an analog-to-digital converter (DI-4108-U, Dataq instruments, Akron, OH, USA).

### Neurovascular coupling protocol with fNIRS

Functional NIRS examination was performed with the NIRScout platform (NIRx Medical Technologies LLC, NY, USA) equipped with 16 light sources and 16 detector optodes. A 128-port Easycap headcap (Easycap GmbH, Woerthsee-Etterschlag, Germany) was positioned to cover the area of the international 10–20 space. The line between Fpz and Iz ports on the headcap was aligned with the sagittal plane of the head, and the optode in the Fpz port was positioned along this line. The cap was set up with stretch-resistant spacers that limit the inter-subject variability of the distance between optodes. The placement of optodes covered the PFC (including DLPFC) and also the medial parts of the motor cortex and somatosensory cortex (Fig 4A, 4D, 4G). Measurements were performed in a quiet and darkened room.

### Data processing and statistical analysis

To assess cognitive performance, reaction time and correct responses (reported as a percentage) obtained from each *n*-back task during the first and second visit were compared using a paired *t*-test (for reaction time) or Wilcoxon signed-rank test (for %correct answers).

For TCD analysis, the mean MCAv was extracted from a 100 second time window from each *n*-back task. To eliminate the effect of motion and additional breathing artefacts, a 10 s period of the recording was disregarded after the initiation of each task. Normal distribution of data was determined with a d’Agostino-Pearson test, and a 2-way repeated measure ANOVA (two factors being the type of cognitive stimulation and the side of MCAv) was performed on the MCAv data followed by a Sidak’s post-hoc test. Increase in MCAv was calculated relative to the 0-back task preceding the 1-back or 2-back task using the following equation: (MCAv_[*n*-back]_-MCAv_[0-back]_)/MCAv_[*0*-back]_*100%, and a paired *t*-test was used to compare the evoked increase in MCAv.

For fNIRS analysis, the raw data were first processed using a pipeline based on a General Linear Model (GLM) approach using the Brain AnalyzIR toolbox (commit 46c645d) [19]. Measured optical densities were converted to hemoglobin concentration using the modified Beer-Lambert law [20], pre-whitening of data was performed with an autoregressive model-based algorithm [21], and a Discrete Cosine Transform based high-pass filter (0.0045 Hz) was used to remove slow drift. Four boxcar regressors (one for each task) were included in the design matrix, which were convolved with a canonical hemodynamic response function to predict brain activation. Beta-weights and scaling of predictors were then used for mixed effect model-based group-level statistics, where a *t*-contrast of [(1-back)—(0-back)], [(2-back)—(0-back)] and [(2-back)—(1-back)] was applied. Increased activation was considered significant when the false discovery rate corrected p<0.05 (FDR using Benjamini-Hochberg method) [22].

To demonstrate the timecourse of HbO and HbR concentration changes, raw fNIRS data were analyzed and processed with nirsLAB (NIRx Medical Technologies, NY, USA) [23]. Saturated channel data and channels with highly variable noise (>7.5% coefficient of variation) were excluded from further analysis. A bandpass filter of 0.0045 Hz to 0.2 Hz was applied to filter physiological noise. Modified Beer-Lambert law [24] was used to convert recorded optical density to change of hemoglobin concentration. differential pathlength factor was adjusted for age with an equation previously described [25]. Block averages were then calculated for each channel during each stimulus, and channel means were then averaged for the region of interest for the two conditions associated with different levels of mental workload (1-back and 2-back conditions). Three regions of interests (ROIs), including medial PFC, left and right DLPFC were selected for plotting [10].

## Results

### Cognitive performance in the group of healthy young adults and the practice effect of the repeated *n*-back task

All subjects scored over 90% correct answers when tested with the cognitive *n*-back task. Reaction times were found to be task-dependent and were significantly longer during the 1-back and 2-back tasks vs. their corresponding 0-back task, on both visits (Table 1).

**Table 1:** Cognitive performance in the group of healthy young adults and the practice effect of the repeated *n*-back task.

	1^st^ trial Median [IQR] or Mean ± SD	2^nd^ trial Median [IQR] or Mean ± SD	*p*-value (t-test or Wilcoxon signed-rank test)
**Reaction time (ms)**			
**0-back #1**	412.6 ± 46.13	407.48 ± 22.6	0.87
**1-back**	531.6 ± 75.56^#^	497.12 ± 35.68^#^	0.37
**0-back #2**	441.7 ± 69.76	451.8 ± 23.66	0.29
**2-back**	644.7 ± 125.11^#^	610.44 ± 38.06^#^	0.5
**Performance (% correct)**			
**0-back #1**	100 [100 to 100]	100 [100 to 100]	>0.99
**1-back**	100 [99.6 to 100]	100 [100 to 100]	>0.99
**0-back #2**	100 [100 to 100]	100 [100 to 100]	>0.99
**2-back**	98.3 [93.3 to 100]	100 [98.3 to 100]	0.06

Cognitive function was measured using *n*-back cognitive task in n = 14 healthy young adults (n = 14). No statistical difference in reaction time and cognitive performance between two visits was observed. Cognitive performance (%correct) is presented as median with interquartile ranges [IQR], and the reaction time is presented as mean±SD.

^#^: Comparison with preceding 0-back task, p<0.01.

To evaluate the practice effect and repeatability of the *n*-back cognitive task, we performed a paired analysis of the cognitive performance (%correct) answers and the reaction time and performance between first and second laboratory visits. We found no significant difference in the performance or the reaction between two visits, however, we observed a trend (p = 0.06) for a minor improvement in the 2-back test performance.

Table 1 reports %correct answers and reaction time for each task of the *n*-back test for each laboratory visit.

### A more challenging cognitive task evokes NVC responses in the MCA measured with TCD

Two participants were excluded from the analysis due to an inadequate temporal acoustic window. Left-sided channel data were excluded in 3 participants, and right-sided channel data were excluded for one participant due to low mean flow velocity or signal loss during testing.

We found no effect of MCAv Side (F(1,18) = 0.6, p = 0.45) or interaction of Cognitive stimulation×MCAv Side (F(3, 48) = 0.61, p = 0.6), however, we found a significant effect of Cognitive stimulation that accounted for 2.54% of variation in the data (F(3, 48) = 8.595, p<0.0001). Post-hoc testing revealed significant differences between the 0-back and 2-back conditions (Table 2, mean difference for left MCAv: 2.65±0.65, t = 4.097, p = 0.0008; right MCAv: 1.71±0.58, t = 2.93, p = 0.0295).

**Table 2:** MCA flow velocity increased with cognitive load.

MCA side and condition	Mean MCAv	Mean Difference	SE of Difference	*t*	*p*
**Left**					
**0-back vs 1-back**	47.85 vs. 48.02	0.17	0.65	0.262	>0.999
**0-back #1 vs 0-back #2**	47.85 vs. 46.58	-1.27	0.65	1.967	0.285
**0-back vs 2back**	46.58 vs. 49.23	2.65	0.65	4.097	<0.001
**RIGHT**					
**0-back vs 1-back**	46.55 vs. 46.44	-0.11	0.65	0.191	>0.999
**0-back #1 vs 0-back #2**	46.55 vs. 45.14	-1.41	0.65	2.418	0.109
**0-back vs 2back**	45.14 vs. 46.85	1.711	0.65	2.928	0.029

Neurovascular coupling hemodynamic responses were measured in the middle cerebral artery (MCA) using transcranial Doppler (TCD) sonography during the performance of the cognitive *n*-back task in healthy young adults (n = 12). Two-way ANOVA revealed no statistically significant effect for the Side of the MCA velocity (MCAv) or the interaction of Cognitive stimulation×MCAv Side. We observed a significant effect of Cognitive stimulation that accounted for 2.54% of variance in the data. Results of the Sidak’s post-hoc test are reported in the table. We observed significantly greater responses in the MCAv during 2-back task vs 0-back task.

**Fig 2:**
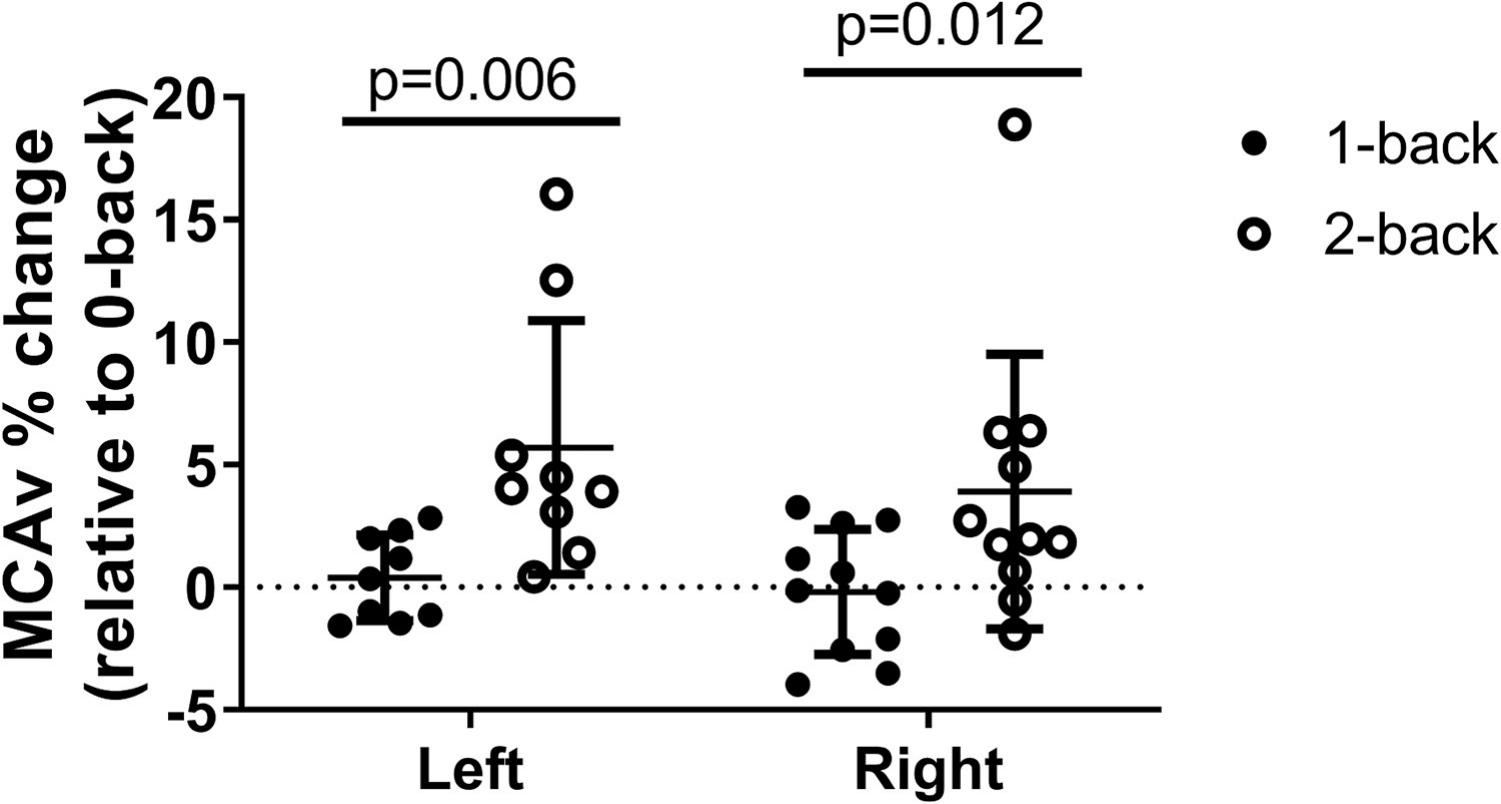
Neurovascular coupling responses measured in the MCA depend on cognitive load. The effect of cognitive load on NVC responses measured using transcranial Doppler sonography (TCD) was evaluated by comparing the percent change in the blood flow velocities between 2-back and 1-back tasks that were normalized to the corresponding 0-back task in n = 12 young and healthy adults. Significantly greater NVC responses were observed during 2-back task compared to 1-back task in both MCAs (left MCA: t = 4.097, p<0.001, right MCA: t = 2.928, p = 0.029).

To evaluate the effect of cognitive workload on NVC responses measured using TCD, we also compared the percent change in the blood flow velocities between 2-back and 1-back tasks that were normalized to the preceding 0-back task. We observed significantly greater NVC responses during 2-back task compared to 1-back task in both MCAs (Fig 2, left MCA: t = 3.73, p = 0.006, right MCA: t = 3.06, p = 0.012).

### A more challenging cognitive task evokes NVC responses in the prefrontal cortex measured using fNIRS

**Fig 3:**
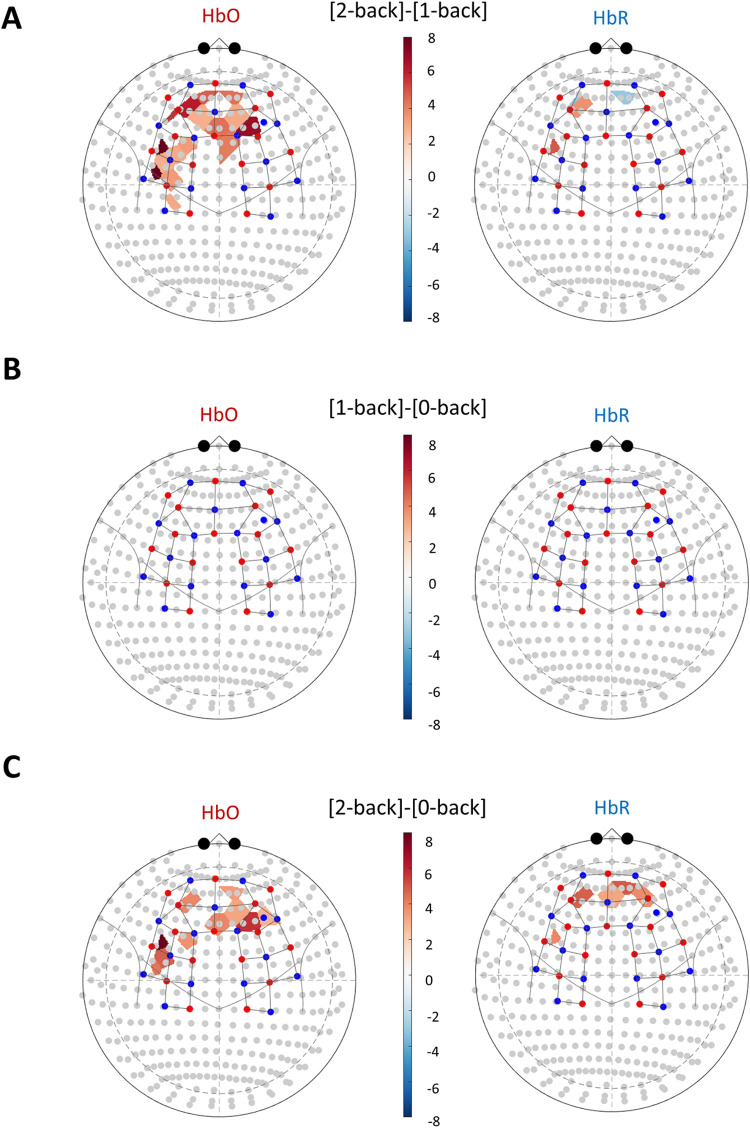
Significant activation of the prefrontal cortex is only detected during the mentally more demanding task with fNIRS. NVC responses were assessed during cognitive *n*-back stimulation using the functional near-infrared spectroscopy (fNIRS) methodology. Tasks were administered in the following order: 0-back→ 1-back→ 0-back→ 2-back, 0-back being the reference condition, 1-back and 2-back being the tasks with different cognitive demand. Blue dots represent detectors and red dots represent light sources of the fNIRS probe. The 3D probe structure and statistically significant different hemodynamic responses were then projected into the international 10–20 space. *t*–values are plotted in a color-coded manner to show differences. We observed a statistically significant increase in the oxy-hemoglobin (HbO) levels in the prefrontal cortex (PFC), an increase in the left dorsolateral prefrontal cortex (DLPFC), and a focal increase over the left motor cortex. Data were analyzed using the Brain AnalyzIR software, and a mask was applied on results to only map channels where *p*_FDR_<0.05. *t*-statistic heatmaps show the significant difference between the more mentally demanding task [2-back] and the less demanding task [1-back] (Panel A). No significant neurovascular coupling response was detected when comparing the [1-back] condition to the baseline condition [0-back] (Panel B). Panel C demonstrates a significant activation during 2-back task when compared to the preceding 0-back task.

**Fig 4:**
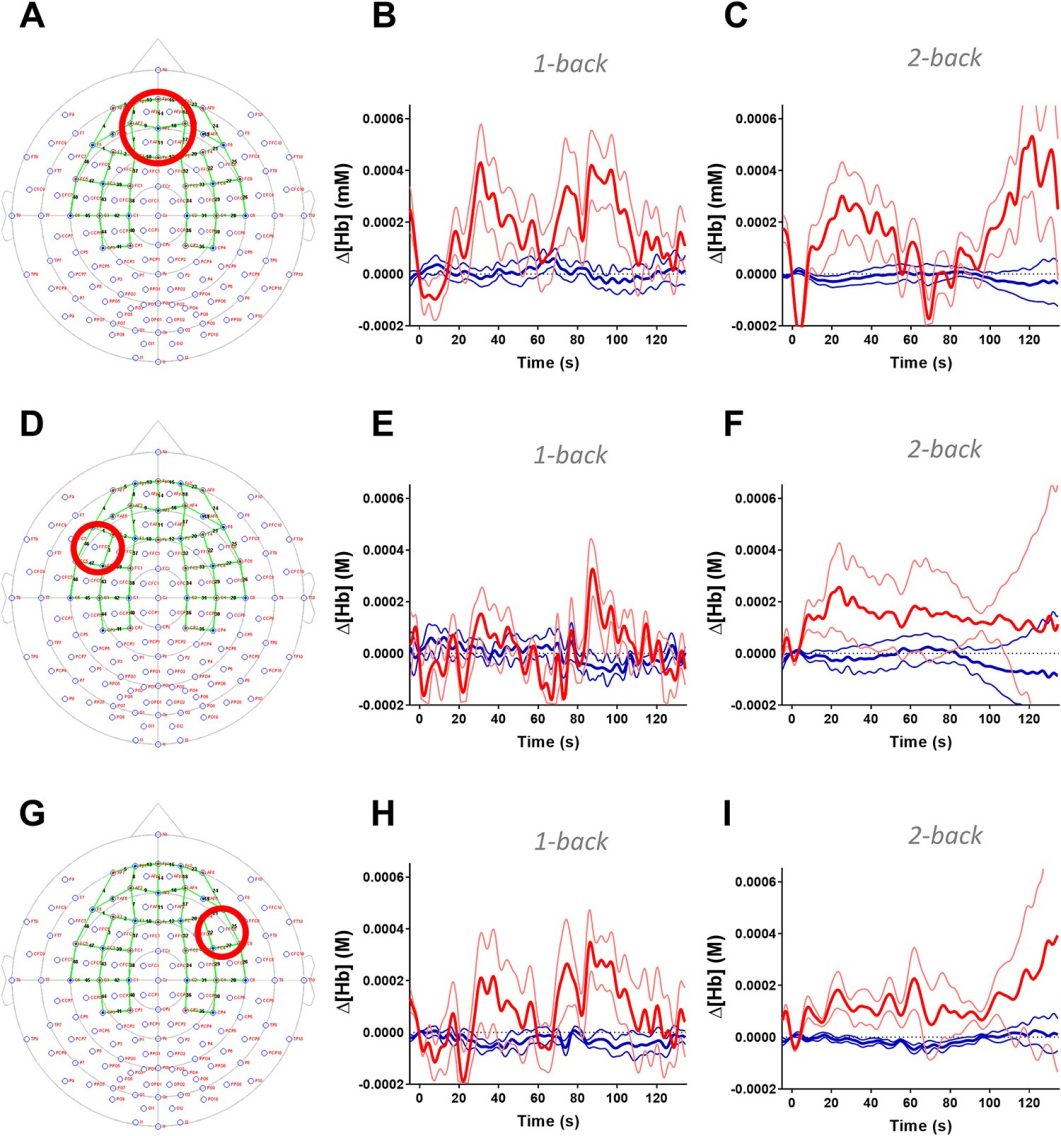
Block average traces over the prefrontal cortex and dorsolateral prefrontal cortex during the cognitive *n*-back stimulation using functional near-infrared spectroscopy. Group average traces of oxy-hemoglobin (HbO, red lines) deoxy-hemoglobin (HbR, blue lines) concentrations were plotted from medial prefrontal cortex (PFC; Panel A), left and right dorsolateral prefrontal cortex (DLPFC; Panel D and G). Region of interests are shown on Panel A, D, and G within the international 10–20 space, and green lines represent channels. 2-back cognitive tasks elicited visible changes in HbO and HbR levels with profound hemodynamic responses during 2-back condition in the medial PFC and left DLPFC (Panel C and F). DLPFC ROI based on Vassena et al. [10]. Mean±SD is plotted for each channel. Data for individual channels are shown in S2 and S3 Figs.

Two participants were excluded from fNIRS analysis due to highly variable noise recorded in >80% of NIRS channels. Cognitive task evoked NVC was evaluated with two different statistical methods when processing the results of fNIRS assessments. The results of the GLM based approach are demonstrated in Fig 3 and S2 Fig. A t-contrast between the 2-back and 1-back task conditions revealed a significant increase in HbO in the PFC, HbO increase in the left DLPFC(channels between 10–20 positions F3-FC3, FC5-FC3 and FC5-F5), and in the left motor cortex (channels FC5-C5, C3-FC3) as shown in the left panel of Fig 3A. Changes in HbR signal were found significant in several channels (Fig 3A, right panel), however, these changes seemed to be bidirectional. We observed both focal increase and a decrease of the HbR concentrations in the prefrontal cortex, in channel AF3-Fp1 and channels Fpz-Fp2, AF7-Fp1, respectively. We also observed an increase in HbR concentrations in the left DLPFC (FC5-F5). When comparing each *n*-back condition to the preceding 0-back condition as a reference, we observed no significant additional response evoked by the 1-back condition (Fig 3B), only the 2-back condition (Fig 4C). The two 0-back conditions were also compared, and minor differences were found in the form of localized inactivation during the second 0-back trial in the prefrontal cortex and the somatosensory cortex (S1 Fig).

Further, to demonstrate the timecourse of NVC responses, we plotted group averaged HbO and HbR timecourses in three regions of interests (ROIs) responsible for executive cognitive function, including the medial PFC, left and right DLPFC (Fig 4A, 4D and 4G). The characteristic hemodynamic response can be seen during the 2-back condition in the medial PFC and prominently in the left DLPFC (Fig 4C and 4F respectively).

## Discussion

The current study compared hemodynamic responses in the brain evoked by a task with different levels of cognitive workload. Cognitive stimulation was based on the *n*-back paradigm, which proved to be a repeatable paradigm to evoke NVC. No significant improvement was seen between visits in the reaction time or performance of subjects during the tasks. The *n*-back performance showed a trend toward improvement; however, the extent of the improvement was minor when measured in participants of the current study. Neither NVC measurements could differentiate NVC responses evoked by the task with lower cognitive workload (1-back) when compared to the baseline (0-back) condition. The more demanding cognitive task (2-back) evoked greater neurovascular coupling responses compared to both the baseline (0-back) and the less demanding task (1-back) when measured in healthy young adults.

During the more difficult task (2-back condition), TCD detected a significant increase in MCA blood flow velocity (Table 2), indicating a downstream dilation of cerebral arterioles.

Functional NIRS is capable of imaging the convexity of the cerebral cortex, and since the MCA supplies the PFC convexity through pial arterioles, it allowed closer investigation of the dilation of blood vessels distal to the MCA. An increase of blood flow to a cortical region is represented by the characteristic increase of HbO and decrease of HbR concentrations in the brain tissues measured by fNIRS [26]. A significant increase of HbO concentration was seen in the medial PFC and left DLPFC during a more challenging cognitive task (2-back versus 1-back and 2-back versus 0-back conditions). When evaluating with the GLM approach, bidirectional changes of HbR signals were recorded with focally increased or decreased activity; however, the used basis function (canonical hemodynamic response function) may not be the ideal model to investigate changes in HbR concentration. When data were evaluated with the block average approach, it is clearly visible that the decrease in HbR concentration is accompanying increases in HbO concentration in the medial PFC and the left DLPFC (Fig 4) when measured during the mentally more demanding condition (2-back). Interestingly, block averaged data showed multiple peaks in the change of HbO and HbR concentrations. This can be a byproduct of the long stimulation period and the mid-stimulation HbO dip may be a residual physiological artefact. The *n*-back paradigm used in this study for both TCD and fNIRS methodologies was originally developed for TCD studies [4, 27], and therefore was not optimized for use with fNIRS assessments. The long stimulation periods did not allow for very strong filtering of fNIRS data to remove low-frequency physiological noise since task-related signals also had a very low frequency. However, this was addressed by performing statistical analysis in a more standardized way with the Brain AnalyzIR toolbox and showing block averaged hemodynamic responses only for demonstrative purposes.

Activation of medial PFC in addition to DLPFC was only significant while participants were presented the more demanding 2-back task. According to earlier neuroimaging studies, the medial PFC (and the dorsal anterior cingulate cortex) is activated when a difficult task was anticipated [28], and these areas partially overlap with areas activated when anticipating a reward [28, 29]. Some studies showed that performing a task correctly may be rewarding on its own, even without any extrinsic incentive [30, 31]. Latter may also help interpret the activation of the medial PFC since participants in this study were encouraged to perform as well as they could. Interestingly, the area of medial PFC during the 2-back task compared to the 1-back task resembles the increase in frontal theta activity assessed by EEG during a similar *n*-back approach [32], which has been described to appear when performing cognitive tasks demanding excessive concentration [33].

Involvement of the DLPFC area has been widely described in working memory [34], which involvement is also further confirmed by other studies where working memory deficits are associated with limited DLPFC activation in patients with schizophrenia [35] or post-traumatic stress disorder [9]. Vassena, E. et al also described DLPFC activation when a mentally challenging task was anticipated [10]. Prior to the administration of the 1-back and 2-back tasks, the trials were cued, which could also further explain the multiple peaks in the HbO and HbR signals (Fig 4). Cueing tasks may lead to an early activation of the PFC during the preparation and the building of strategy for the tasks, and the late activation peak could be evoked by performing the task itself.

To summarize, hemodynamic NVC responses were successfully assessed using fNIRS and TCD with the same cognitive stimulation paradigm after reaching a certain level of difficulty. The combination of the two NVC assessments allowed better understanding of the cerebral hemodynamics in healthy individuals, which also means that presented procedures can potentially detect cerebrovascular dysfunction. Our data suggest that if NVC function is to be monitored, utilizing the more ubiquitous TCD may be sufficient if the stimulation paradigm and task difficulty are carefully selected. On the other hand, inference from purely TCD results may be limited as it does not differentiate between the spatiotemporal pattern of cerebral hemodynamic changes. Additional cortical areas might be recruited, or the timecourse and amplitude of hemodynamic response may change depending on the task difficulty level in the cortical area that was expected to be activated.

Due to limited metabolic reserve, intact neuronal and glial function underlying brain activity depends on constant provision of adequate oxygen and nutrient supply via continuous adjustment of cerebral blood flow. The importance of NVC is evidenced by the fact that disruption of NVC in a rodent model leads to the impairment of neuronal function, and consequently, cognitive impairment [36]. Further, growing evidence suggests that cerebromicrovascular impairment plays a causal role in the development of cognitive impairment [37]. Studies assessing NVC with classic neuroimaging methods (e. g. fMRI) often consider NVC only as a marker of neuronal activation, its amplitude proportional to the extent of neuronal activation, and disease-related change in NVC is rarely considered. On the other hand, studies utilizing TCD to assess NVC more commonly consider NVC a phenomenon that is dependent on cardiovascular disease and age [4]. Therefore, understanding the NVC responses in human subjects and developing appropriate methodologies and stimulation paradigms opens opportunities to investigate the NVC as a mechanism for mild cognitive impairment in aging, Alzheimer’s disease, and other diseases that are associated with cognitive decline. Further, improving NVC responses, as it has been demonstrated in animal models of aging and age-related diseases [38–42], can also serve as a potential target for development interventions to battle cognitive decline in aging and other age-related diseases. Finally, it is important to highlight the limitations of the current study. The fNIRS technology only allows recording hemodynamic responses from the convexity of the PFC, therefore, dorsal anterior cingulate cortex and the striatum cannot be examined with this method. Measurement of these areas would allow better insight into the neuronal resource recruitment process, possibly explaining the multiple peaks in HbO and HbR signals observed, as contribution of these areas to preparing and estimating the cost/benefit of a certain task has been described [29–31]. This study also did not investigate the effects of motivation on the evoked NVC responses, therefore, we assumed similar motivation levels in all participants, as they were all motivated by the examiner the same way. The method of cognitive stimulation used in this study is designed to detect an age-related decline in working memory [4, 14], and in this study we examined healthy young individuals. Due to the observed high performance of enrolled subjects, we could not examine the association of impaired NVC to impaired working memory performance.

## Conclusions

The current study demonstrates the sensitivity of fNIRS and TCD to differentiate between NVC responses evoked by cognitive stimulation with different levels of cognitive workload. In healthy adults, only a more challenging working memory task evoked significantly greater NVC responses both in the left DLPFC and medial PFC regions, as well as in the MCA supplying these regions.

## Supporting information

S1 Fig NVC responses were assessed during cognitive *n*-back stimulation using the functional near-infrared spectroscopy (fNIRS) methodology. Tasks were administered in the following order: 0-back→ 1-back→ 0-back→ 2-back, 0-back being the reference condition, 1-back and 2-back being the tasks with different cognitive demand. Significant inactivation was seen in areas of the prefrontal cortex (PFC) and left somatosensory cortex when the first reference 0-back condition was compared to second 0-back condition. Data were analyzed using the Brain AnalyzIR software, and a mask was applied on results to only map channels where *p*_FDR_<0.05. T-statistic heatmaps are plotted.
(TIF)

S2 Fig Block average traces over the prefrontal cortex and dorsolateral prefrontal cortex during the cognitive *n*-back stimulation using functional near-infrared spectroscopy (NIRS). Group average traces of oxy-hemoglobin (HbO) for each channel of the NIRS probe. Red lines represent the channels regions of interest defined in Fig 4, and grey lines are other channels in the area named above each panel.
(TIF)

S3 Fig Block average traces over the prefrontal cortex and dorsolateral prefrontal cortex during the cognitive *n*-back stimulation using functional near-infrared spectroscopy (NIRS). Group average traces of deoxy-hemoglobin (HbR) for each channel of the NIRS probe. Blue lines represent the channels in the regions of interest defined in Fig 4, and grey lines are other channels in the area named above each panel.
(TIF)
