# Supplementary figures and images for: Increased cognitive workload evokes greater neurovascular coupling responses in healthy young adults
Source: PLoS One. 2021 May 19;16(5):e0250043. doi: 10.1371/journal.pone.0250043 (PMC8133445; doi:10.1371/journal.pone.0250043)

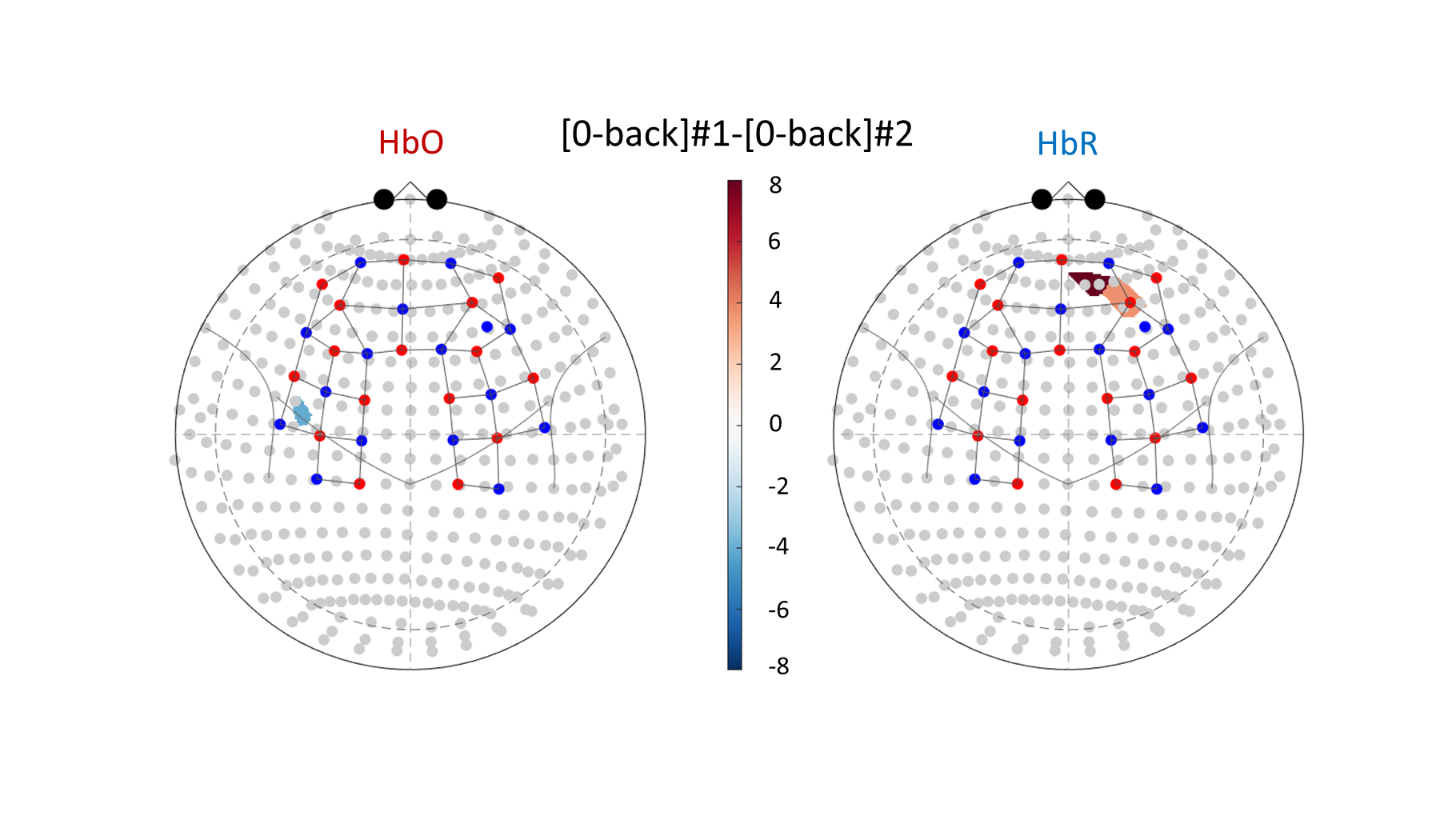

Supplement: S1 Fig — Tasks were administered in the following order: 0-back→ 1-back→ 0-back→ 2-back, 0-back being the reference condition, 1-back and 2-back being the tasks with different cognitive demand. Significant inactivation was seen in areas of the prefrontal cortex (PFC) and left somatosensory cortex when the first reference 0-back condition was compared to second 0-back condition. Data were analyzed using the Brain AnalyzIR software, and a mask was applied on results to only map channels where pFDR<0.05. T-statistic heatmaps are plotted. (TIF) [file pone.0250043.s001.tif]

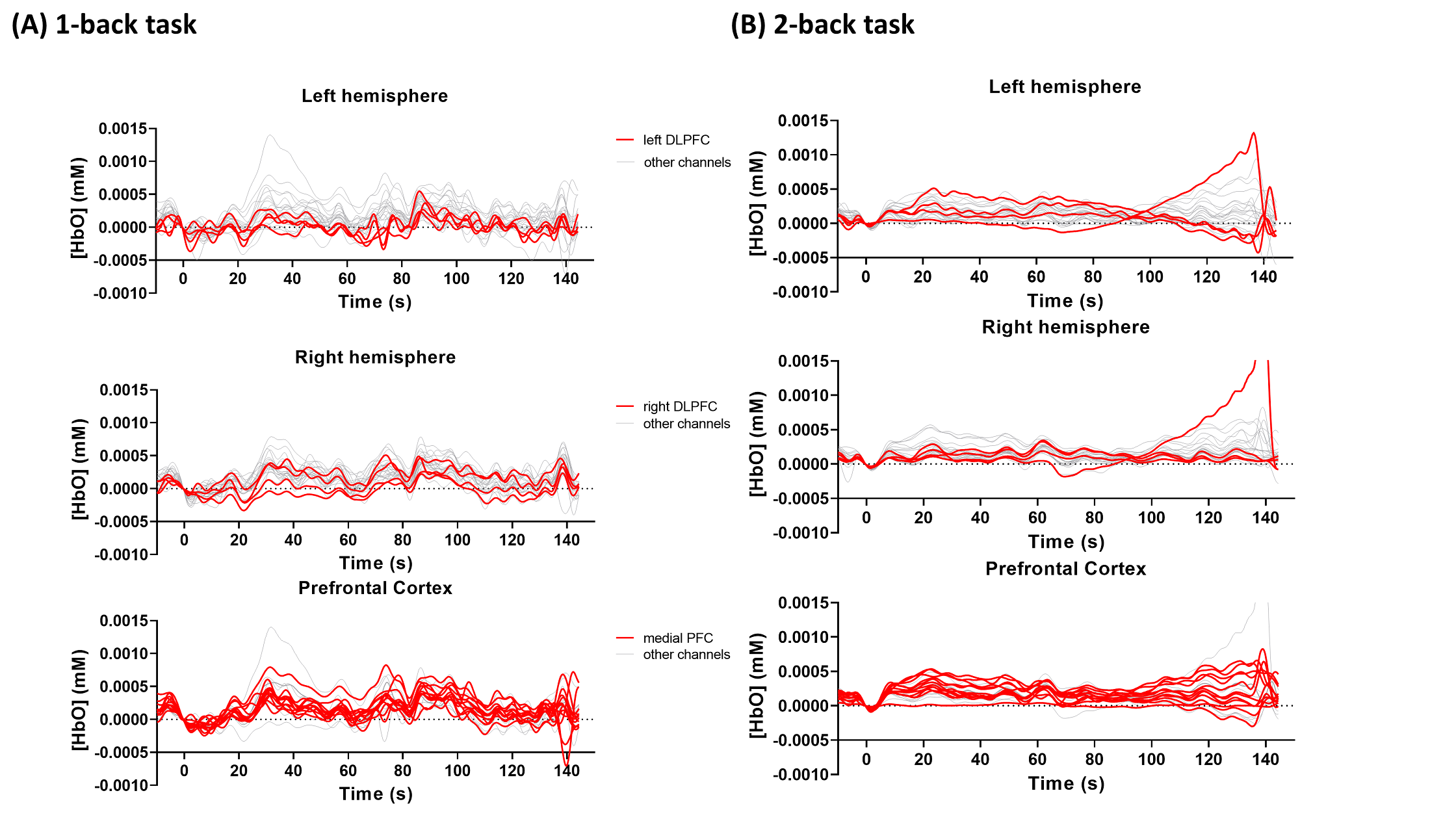

Supplement: S2 Fig — Group average traces of oxy-hemoglobin (HbO) for each channel of the NIRS probe. Red lines represent the channels regions of interest defined in Fig 4, and grey lines are other channels in the area named above each panel. (TIF) [file pone.0250043.s002.tif]

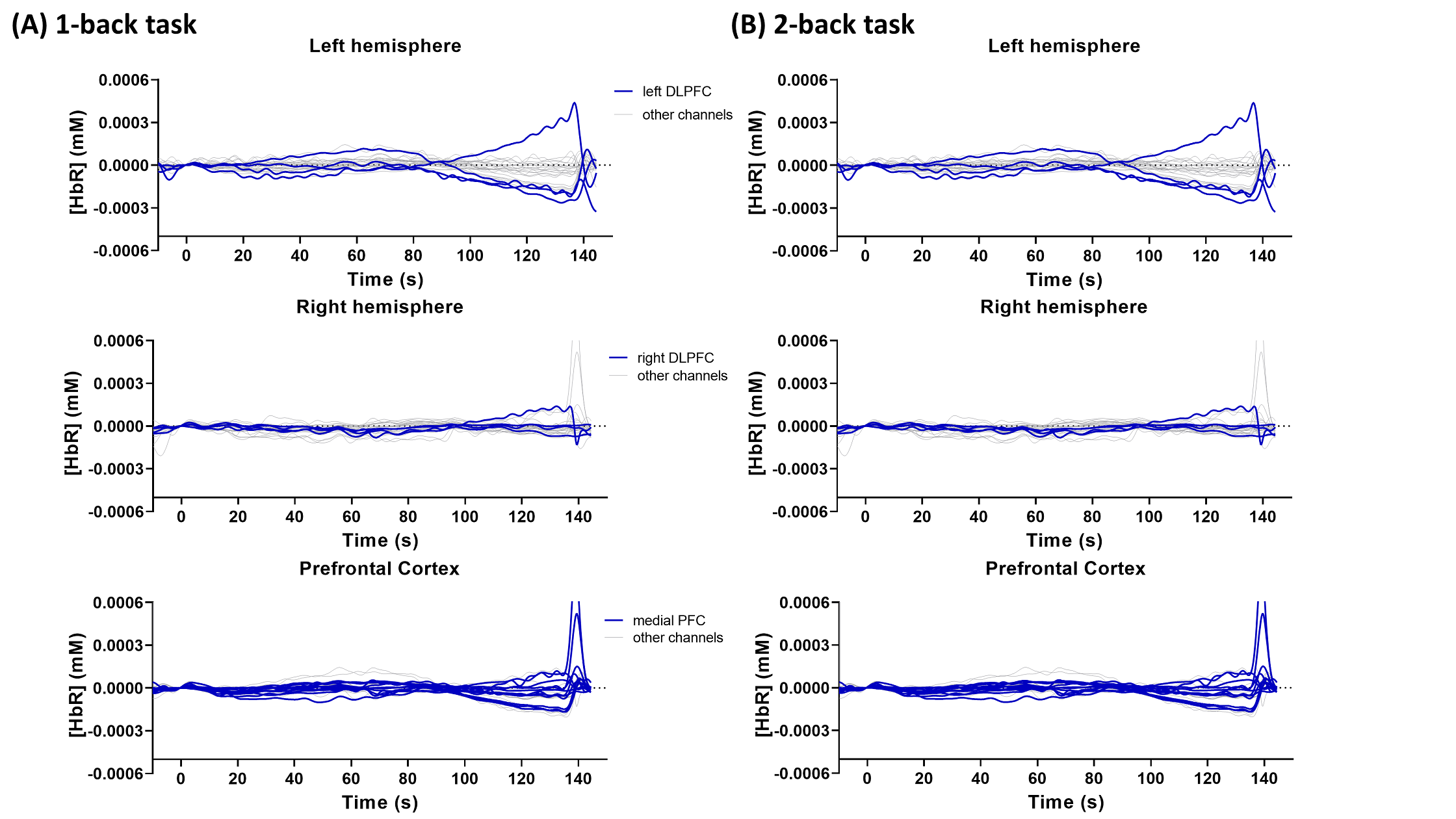

Supplement: S3 Fig — Group average traces of deoxy-hemoglobin (HbR) for each channel of the NIRS probe. Blue lines represent the channels in the regions of interest defined in Fig 4, and grey lines are other channels in the area named above each panel. (TIF) [file pone.0250043.s003.tif]
